# A One-Pot Synthesis and Characterization of Antibacterial Silver Nanoparticle–Cellulose Film

**DOI:** 10.3390/polym12020440

**Published:** 2020-02-13

**Authors:** Qi-Yuan Chen, Sheng-Ling Xiao, Sheldon Q. Shi, Li-Ping Cai

**Affiliations:** 1 College of Engineering and Technology, Northeast Forestry University, 26 Hexing Road, Harbin 150040, China; cqy454145940@gmail.com; 2 Department of Mechanical and Energy Engineering, University of North Texas, Denton, TX 76203, USA; Sheldon.Shi@unt.edu (S.Q.S.); Liping.Cai@unt.edu (L.-P.C.)

**Keywords:** silver nanoparticles, one-pot method, cellulose film, antibacterial

## Abstract

Using N,N-dimethylacetamide (DMAc) as a reducing agent in the presence of PVP-K30, the stable silver nanoparticles (Ag-NPs) solution was prepared by a convenient method for the in situ reduction of silver nitrate. The cellulose–Ag-NPs composite film (CANF) was cast in the same container using lithium chloride (LiCl) giving the Ag-NPs-PVP/DMAc solution cellulose solubility as well as *γ*-mercaptopropyltrimethoxysilane (MPTS) to couple Ag-NPs and cellulose. The results showed that the Ag-NPs were uniformly dispersed in solution, and the solution had strong antibacterial activities. It was found that the one-pot synthesis allowed the growth of and cross-linking with cellulose processes of Ag-NPs conducted simultaneously. Approximately 61% of Ag-NPs was successfully loaded in CANF, and Ag-NPs were uniformly dispersed in the surface and internal of the composite film. The composite film exhibited good tensile properties (tensile strength could reach up to 86.4 MPa), transparency (light transmittance exceeds 70%), thermal stability, and remarkable antibacterial activities. The sterilization effect of CANF_0.04_ against *Staphylococcus aureus* and *Escherichia coli* exceed 99.9%. Due to low residual LiCl/DMAc and low diffusion of Ag-NPs, the composite film may have potential for applications in food packaging and bacterial barrier.

## 1. Introduction

Silver nanoparticles (Ag-NPs) refer to silver clusters with a particle size of 1 to 100 nm, which possess a large specific surface area and have a good inhibitory effect against Gram-positive bacteria, Gram-negative bacteria, fungi, Pseudomonas, and bacteriophages [1]. Silver nanoparticles with different particle sizes present varied toxic effects on bacteria. Studies have found that Ag-NPs with a size of 5–20 nm had greater antibacterial activities [2,3,4]. Currently, the widely recognized antibacterial mechanisms of Ag-NPs include disrupting the normal function of the cell wall [5], interacting with the lipid components of the cell membrane to impede its normal function [6,7,8,9,10], inducing ROS free radicals to damage the cell membrane [11,12,13], damaging the DNA structure and inhibiting its related functions [14,15,16], binding with sulfhydryl groups of enzyme proteins to make cell inactive, and so on [17,18]. Ag-NPs inhibits the characteristics of simple preparation, broad-spectrum antibacterial, strong sterilization, and less prone to emerge drug resistance, which prompted it to be used as an antibacterial agent added to ceramics [19], coatings [20], textiles [21], films [22,23], and other raw materials to fabricate antibacterial materials.

Cellulose molecules have active hydroxyl groups that can be combined with other polymers, inorganics, organics, and nanomaterials. It expresses the characteristics of environmentally friendly, cheap, easy to obtain, and facile film formation abilities [24]. To make cellulose own antibacterial activities, blending Ag-NPs with cellulose forming to antibacterial films is a research hotspot in related fields [25]. Recently, there was an increasing research interest in the preparation of cellulose Ag-NPs composite films used in situ generation, which immersed the cellulose film in an aqueous solution containing Ag^+^. When Ag^+^ was fully immersed into the film, the reducing agent was used to reduce the Ag^+^ and precipitate to obtain silver loaded composite films. reducing agents, such as glucose and sodium borohydride, are widely used [26,27,28]. This method can be used to prepare a composite film with good antibacterial properties and relatively stable Ag-NPs adsorbed on the film surface. However, the experimental process requires a large amount of Ag to immerse the film, which would cause a mass of silver waste. Blending Ag-NPs with cellulose is one of the traditional methods to prepare silver-loaded composite films. The experimental process is uncomplicated and without silver waste [29]. However, Ag-NPs have poor compatibility with cellulose and are liable to form partial agglomeration during the film preparation, which results in the size range expansion of Ag-NPs and uniformly distributed in the film [30].

To solve this problem, we put forward a pre-reaction that reduces Ag^+^ with in situ generation in N,N-dimethylacetamide (DMAc) under the protection of polyvinylpyrrolidone (PVP) for short time to form a small size of Ag-NPs/PVP solution. Cellulose was dissolved in the solution as Ag-NPs continue to grow, aiming to enhance the dispersibility of Ag-NPs within cellulose and the solution. And the mixture was further cast to silver loaded cellulose composite film. Only one reagent (DMAc) was used in the experiment, and all reactions can be completed in a single container, which reduces the cost of the reagents and without complex operation. The morphology and chemical structure of the Ag-NPs were characterized by Fourier transform spectroscopy (FT-IR), X-ray diffraction (XRD), and transmission electron microscope (TEM). The surface morphology and chemical structure of the composite film were evaluated by scanning electron microscopy (SEM), thermogravimetric analysis (TGA), X-ray photoelectron spectroscopy (XPS), XRD, gas chromatography–mass spectrometer (GC-MS), inductive coupled plasma emission spectrometer (ICP), light transmittance and haze detection, and tensile tests. The minimum inhibitory concentration (MIC), minimum bactericidal concentration (MBC) value, inhibition zone, and contact antibacterial ability of the fabricated films against *Escherichia coli* (*E. coli*) and *Staphylococcus aureus* (*S. aureus*) were investigated to provide further research with theoretical supports.

## 2. Materials and Methods 

### 2.1. Materials

Cellulose (*α*-cellulose >80%) was experimentally self-made via the preparation method described in previous experiments [31]. *γ*-Mercaptopropyltrimethoxysilane (MPTS) was provided by Macklin Biochemical Technology Co., Ltd. (Shanghai, China). Silver nitrate solid was purchased from Haiyinpeng Chemical Trade Co., Ltd. (Tianjin, China). Polyvinylpyrrolidone-K30 (PVP), lithium chloride (LiCl), N,N-dimethylacetamide (DMAc; with a moisture content of less than 0.1%), and other analytical reagents used in the experiment were purchased from Fuyu Fine Chemicals Co., Ltd. (Tianjin, China). *S. aureus* (ATCC 6538) and *E. coli* (ATCC 25922) were obtained from China General Microbiological Culture Collection Center (Beijing, China).

### 2.2. Preparation of Ag-NPs Solution in Organic Phase

First, we weighed 0.0630 g of AgNO_3_ (contain 0.04 g of silver equivalent) into a 150 mL flat bottom flask. Then, we added 0.3 g of PVP and 45 g of DMAc to the flask and shook thoroughly until the solution turned brown. The solutions were placed in the dark room at 40 °C for 1 h and 4 h, respectively, to obtain organic Ag-NPs solution with different reaction degrees. We weighed 0.3 g of PVP and 45 g of DMAc to a flask as a blank control solution. Solutions were used to characterize the morphology, chemical structure, and antibacterial activity of Ag-NPs.

We eeighed 0 g, 0.0079 g, 0.0158 g, 0.0315 g, 0.0630 g, and 0.1260 g of AgNO_3_ (containing 0 g, 0.005 g, 0.01 g, 0.02 g, 0.04 g, and 0.08 g of silver equivalent, respectively) into a 150 ml flat-bottom flask. We added 0.3 g of PVP and 45 g of DMAc to the flask and shook thoroughly until the solution turned brown. The solution was placed in the dark room at 40 °C for 1 h, to obtain organic Ag-NPs solutions with different silver contents. The solution was used for further film fabrication. 

### 2.3. Preparation of Silver Loaded Cellulose Films

We took out the solutions with different silver contents, added 50 μL of MPTS, and stirred in a 50 °C oil bath for 4 h to prepare the coupled Ag-NPs solutions. We accurately weighed 4 g of LiCl solids into the solution and stirred at 105 °C until the solids were dissolved. LiCl was added to form a 8% LiCl/DMAc system. Note that LiCl/DMAc as a widely used cellulose solubilizing system, and LiCl associated with DMAc to form a special Li^+^(DMAc)Cl^-^ structure providing the Ag-NPs solution with cellulose solubility. The major function of this system is to break the most hydrogen bonds of cellulose structures and thereby disperse cellulose in solution [32]. One gram of cellulose was then added to the solutions and continuously heated and stirred for 2 h. The obtained mixtures were cooled to room temperature and remained for 12 h to eliminate bubbles. Then, the mixtures were uniformly cast onto glass plates to pre-solidify them in air. The solidified gels were immersed in the distilled water bath at 20 °C for 4 h and ultrasound for 10 min to remove the residual solvent. We took out the wet cellulose hydrogel samples and dried them at room temperature to obtain uniform films. The film cast by the solutions without Ag-NPs was regenerated cellulose film, labeled as RCF. The cellulose-Ag-NPs films cast by the solutions contain 0.005 g, 0.01 g, 0.02 g, 0.04 g, and 0.08 g of silver and were labeled as CANF_0.005_, CANF_0.01_, CANF_0.02_, CANF_0.04_, and CANF_0.08_, respectively. 

### 2.4. Morphology and Chemical Structure of Ag-NPs

The micromorphology and size of the Ag-NPs were observed via TEM (JEM-2100; JEOL, Tokyo, Japan) at acceleration voltage of 80 kV. The droplets of Ag-NPs solution at reaction time of 1 h and 4 h were dropped on the carbon-coated electron microscopy grids and measured directly. The sizes of Ag-NPs were measured from the TEM images using a Nano Measurer software. 

The FT-IR spectra of reacted 4 h of Ag-NPs solution and PVP blank solution were collected on a Frontier instrument (Frontier, PerkinElmer, CA, USA) in the range from 400 cm^−1^ to 4000 cm^−1^ with a resolution of 4 cm^−1^. The samples were pretreated by quick vacuum drying at 20 °C to form the thick films, following by total reflection scanning. 

An XRD (X` Pert3 Powder, PANalytical B.V., Amsterdam, Netherlands) with Ni-filtered Cu Kα radiation was utilized at an operating voltage of 40 kV and current of 30 mA to analyze the crystal structure of Ag-NPs and PVP thick film samples. The diffraction intensities were collected between 2*θ* = 5° to 80° at a scanning rate of 4°/min. The relative average particle size (*D*) of samples was estimated using the Scherrer formula, as shown in Equation (1):

(1) $$D=\frac{K\gamma }{B\cos\theta },\;$$

where *D* is the average thickness of the crystal grains perpendicular to the crystal plane, which is used as the average particle size of Ag-NPs; *K* is the Scherrer constant with a value of 0.89; B is the full width at half maximum (FWHM) of the diffraction peak; *θ* is the Bragg diffraction angle; and *γ* is X-ray wavelength with a value of 0.154056.

### 2.5. Surface Morphology and Properties of Composite Films

The surface and cross-section micromorphology of RCF and CANF_0.04_ were observed via SEM (Quanta200; FEI Company, Golden, CO, USA) at low acceleration voltages of 12.5 kV. The films were coated with gold by a vacuum sputter coater (SCD 005; Bal-tec™, Los Angeles, CA, USA) before observation. XRD was used to measure and calculate the average particle size of Ag-NPs and cellulose crystallinity in the films. The cellulose crystallinity was calculated by the ratio of the crystalline area to the amorphous area of the samples.

The C, O, and Ag content of the RCF and CANF_0.04_ surface was measured using XPS scanning (THERMO, Thermo Fisher Scientific, Tewksbury, MA, USA). The scanning method performed full scan of the samples (resolution: 1 eV), and accurately scan the C1s and Ag3d peak areas of the samples (resolution: 0.1 eV). 

The main residuals in the film samples obtained from the experiments were DMAc and Li element. The DMAC residues in RCF and CANF_0.04_ were studied by GC/MS (7890A-7000B, Agilent, Palo Alto, CA, USA) with Agilent 19091S-433 column and EI ion source. The test method was according to the SN standard SN / T 3587-2016. The safe residual dose of DMAc was based on OEKO-TEX certification Standard100. A 100 μg/mL DMAc standard solution was used as the threshold to determine whether the solvent content exceeded the standard. The Li residuals in RCF and CANF_0.04_ films were studied by ICP (Optima8300, Agilent, Palo Alto, CA, USA). One gram of RCF and CANF_0.04_ were digest it in 20 mL of 72% concentrated sulfuric acid, respectively. Added 20 mL of 64% nitric acid until the solution becomes clear, and set volume to 1 L with deionized water for measuring.

According to the ISO 1924-2 (2008) standard, mechanical property tests were conducted at room temperature using a universal testing machine (IMT-202F, International Material Tester Co., Ltd., Dongguan, China). The cross-head speed was 10 mm/min. The dimensions of the test films were 100 mm × 10 mm × 0.02 mm to 0.04 mm. The initial separation of the grips was 50 mm.

The light transmittance and haze of the RCF and CANF_0.04_ were measured using a light transmittance/haze meter (WGT-S, Thermo Fisher Scientific, Tewksbury, MA, USA). The light source was a standard light source C, and the films selected for testing were thickness of 0.03 mm. 

The thermal stability of RCF and CANF_0.04_ was measured by TGA using a TG analyzer (SDT Q600; TA instruments, New Castle, DE, USA) at a heating rate of 20 °C/min from 50 °C to 750 °C under a high purity air atmosphere. 

### 2.6. Antibacterial Activities of Ag-NPs and Composite Films

The antibacterial activity of Ag-NPs against *E. coli* and *S. aureus* was determined by MIC and MBC values. Both MIC and MBC were measured according to the Clinical and Laboratory Standards Institute standards CLSI M07-A10 (2015) and CLSI M26-A (1999). 

The antibacterial activity of composite films against two kinds of bacteria was qualitatively tested by the film inhibition zone; 0.5 mL of 10^6^ CFU/mL fresh bacterial bacteria solution was evenly coated on the nutrient agar (NA) plate in the clean bench. Then, 13 mm diameter of round punched RCF to CANF_0.08_ was attached to the surface of the plate. The plates were incubated at 37 °C for 48 h, and the inhibition zone diameter of the films was simply measured with a vernier caliper.

The contact sterilization ability of silver loaded cellulose films against the two types of bacteria was determined according to the ISO 22196 (2011) standard. Approximately 10^6^ CFU/mL of fresh bacterial bacteria solution was coated on the surface of the blank plate (RCF and CANF_0.04_), and covered with polypropylene films. After incubation at 37 °C for 24 h, the plate, film, and cover film were washed carefully using 20 mL nutrient broth (NB)/H_2_O eluent with a volume ratio of 1:100 (v/v); 0.5 mL of the eluate was then coated on the NA plate and incubated for 48 h at 37 °C with a humidity of >95% before the growth of bacteria was observed.

## 3. Results and Discussion

### 3.1. Analysis of Ag-NPs Morphology and Chemical Structure

**Figure 1:**
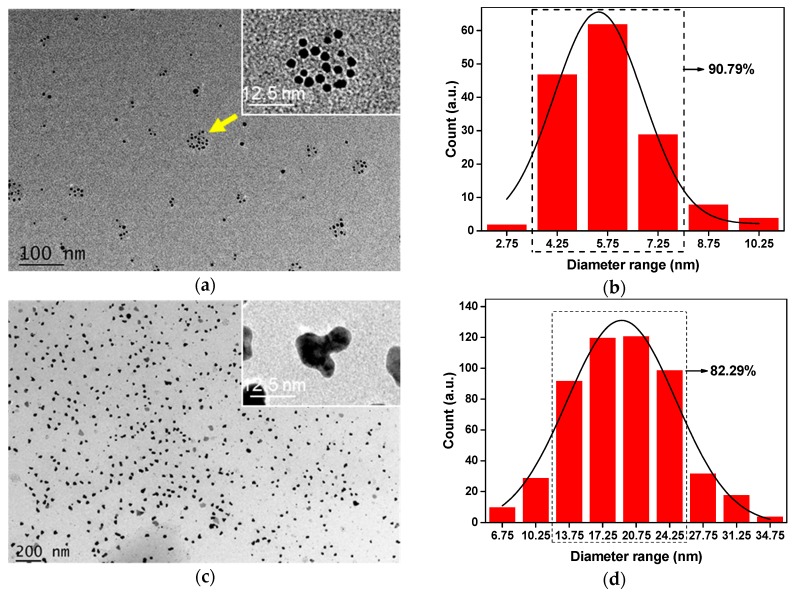
Morphology of Ag-NPs: (**a**,**b**) TEM graph and particle size distribution of Ag-NPs reacted 1 h; (**c**,**d**) TEM graph and particle size distribution of Ag-NPs reacted 4 h.

DMAc possess low ionization energy and electron affinity, which has a weak ability to reduce silver ions, resulting in low rate of silver nanoparticles form to crystal nuclei, and thus the small size Ag-NPs can be prepared [33]. After one hour of reaction, the size of 90.79% of Ag-NPs was approximately 3 to 8 nm in the solution (Figure 1a,b). Smaller Ag-NPs formed into clusters quickly, but not agglomerated to large particles, demonstrating that PVP as a surfactant effectively prevent the rapid agglomeration of Ag-NPs. The size distribution of 82.29% Ag-NPs was 13 to 28 nm (Figure 1c,d), showed that the increase of the reaction time lead to part of Ag^+^ continues to be reduced in situ in existing nanoparticles, deepen the austenitic maturity of Ag-NPs [34], thereby the Ag-NPs size enlarged. The distribution curve of Ag-NPs reacted for 4 h was closer to the normal distribution, and the amount of silver nanoparticles increased remarkably within the visible range, indicating that more Ag^+^ was reduced to Ag-NPs. When PVP formed a coating on the surface of Ag-NPs, the agglomeration process effectively reduced, thus the size of the silver nanoparticles was stabilized within a certain range and closer to the normal distribution. 

**Figure 2:**
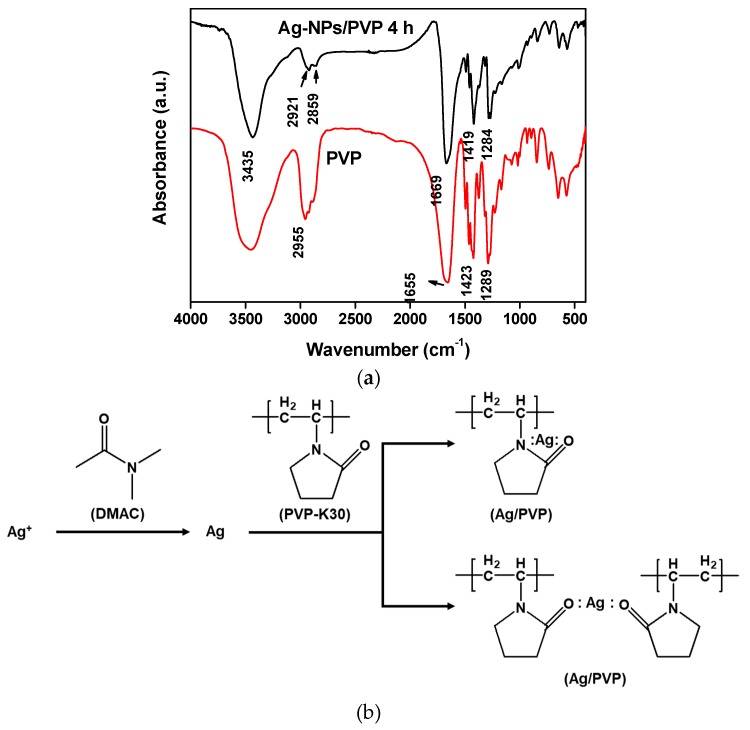
Chemical structure of Ag-NPs/PVP: (**a**) FTIR spectra of PVP and Ag-NPs/PVP reacted for 4 h; (**b**) the major reaction scheme of Ag/PVP.

The FTIR spectrums (Figure 2a) showed the PVP stretching vibration peak of –OH at approximately 3430 cm^−1^ and the stretching vibration peaks of –CH at approximately 2950 cm^−1^ in both lines. The C=O stretching vibration peaks at PVP spectrum was 1655 cm^−1^, whereas the peak of silver was slightly blue shift in Ag-NPs/PVP, which is similar to that described in related literature [35]. This suggest the coordination of O atom in PVP and Ag atom on the surface of Ag-NPs, leading to blue shift of the PVP absorption peak to 1669 cm^−1^ (shown in Figure 2b). The lactam group of PVP at 1423 cm^−1^ and 1289 cm^−1^ were slightly red-shifted when blended with Ag-NPs, the reason was N atom on the lactam group coordinated on the empty orbital of the Ag atom. The existence of the chemical bonding N:Ag:O and O:Ag:O within the PVP makes the Ag-NPs/PVP stable and hard to agglomerate [36].

**Figure 3:**
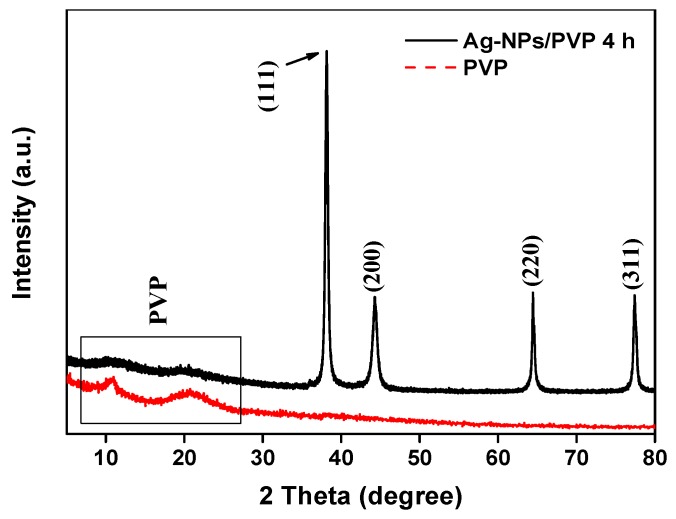
XRD patterns of PVP and Ag-NPs reacted for 4 h.

The XRD patterns (Figure 3) showed 2*θ* at approximately 22° were the diffuse peaks of PVP and Ag-NPs/PVP samples. The diffraction peaks at 2*θ* = 38°, 44°, 64.3°, and 77.3° in Ag-NPs/PVP curve correspond to the face-centered cubic (FCC) silver plane (111), (200), and (220), respectively. The average crystal size of Ag-NPs estimated by Scherrer formula was 14.88 nm, which was consistent with the actual silver nanoparticles size range (13–28 nm) observed by TEM. The analysis results show that the small size Ag-NPs protected by PVP exist stably in DMAc solution.

### 3.2. Analysis of Films’ Surface Morphology and Properties

**Figure 4:**
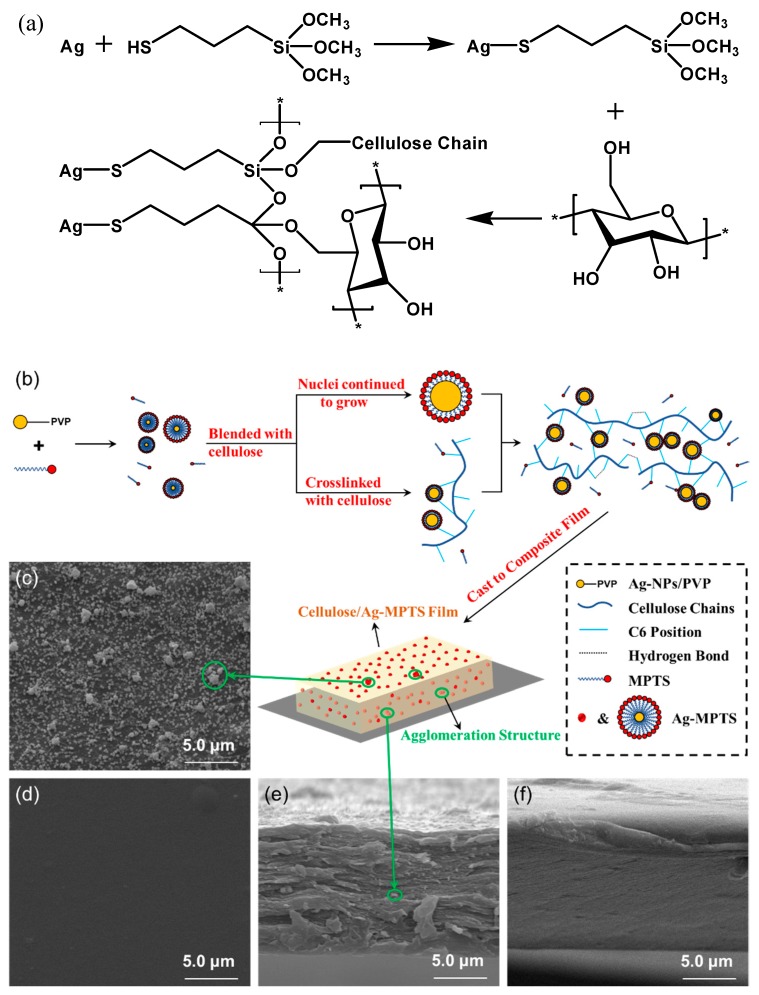
Reaction process and surface morphology of films: (**a**) Reaction scheme of Ag-MPTS-Cellulose cross-linked structure; (**b**) Reaction process of films; (**c**,**e**) SEM graphs of RCF and CANF_0.04_ surface; (**d**,**f**) SEM graphs of RCF and CANF_0.04_ cross-section.

**Figure 5:**
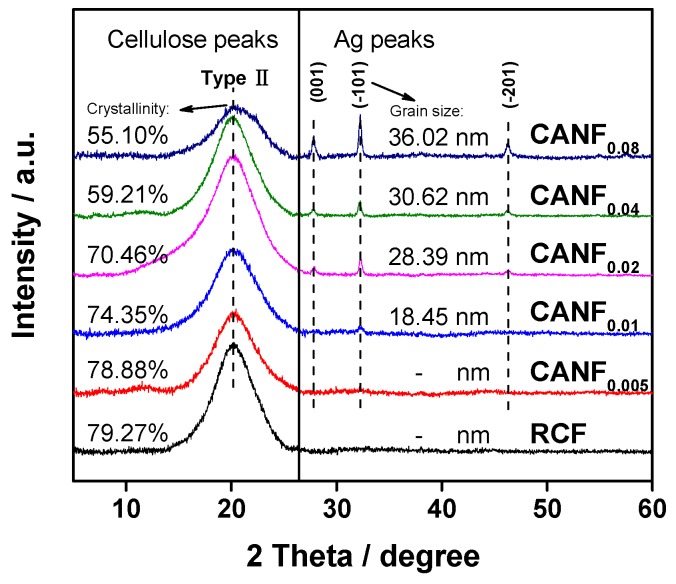
XRD patterns of composite films.

The scheme of Ag-NPs growth and cross-link with cellulose in film-forming process were summarized as shown in Figure 4b. The average size of Ag-NPs calculated by XRD patterns (Figure 5) is larger than the average particle size of Ag-NPs in Ag-NPs/PVP solution, demonstrating that Ag-NPs continued to grow within film formation process. The –SH group in MPTS reacted with Ag to form a better bonding structure (Figure 4a), and the siloxane group of MPTS was bonded to cellulose [37,38]. The chemical bonding effect of MPTS makes cellulose and Ag-NPs more easily cross-linked. It can be seen that some nanoparticles exist on the cross-section of the CANF_0.04_ compared with RCF (Figure 4e,f). These particles combined with cellulose forming a multilayer Ag-MPTS-Cellulose cross-linked structure, and there were some of hydrogen bonds between cellulose chains that were not broken by the cross-linked structure. Therefore, the two processes of Ag-NPs growth and cross-linking with cellulose during the one-pot synthesis were conducted simultaneously. Ag-NPs-MPTS existed on the surface and partly formed an agglomeration structure. This can be proven because the RCF surface was smooth, whereas a mass of spherical nanoparticles appeared on the CANF_0.04_ surface (Figure 4c,d), and some of them agglomerated into larger particles. However, a uniform cellulose-Ag-NPs composite film was successfully prepared before Ag-NPs-MPTS agglomerated excessively. 

The crystal lattice of the obtained cellulose films was type II (Figure 5), and more silver crystal peaks appeared in the spectrum. The average particle size of the Ag-NPs in CANF_0.01_ to CANF_0.08_ was increased by calculation, whereas the cellulose crystallinity was declined. There is a correlation between the two tendencies: the increase of Ag-NPs concentration in film fabrication process caused the nanosilver particles more easily to agglomerate, and the average particle size increased. In addition, more uniform filling Ag-NPs within cellulose structures may affect the cellulose crystallization ability, resulting in a decrease in cellulose crystallinity of the films.

**Figure 6:**
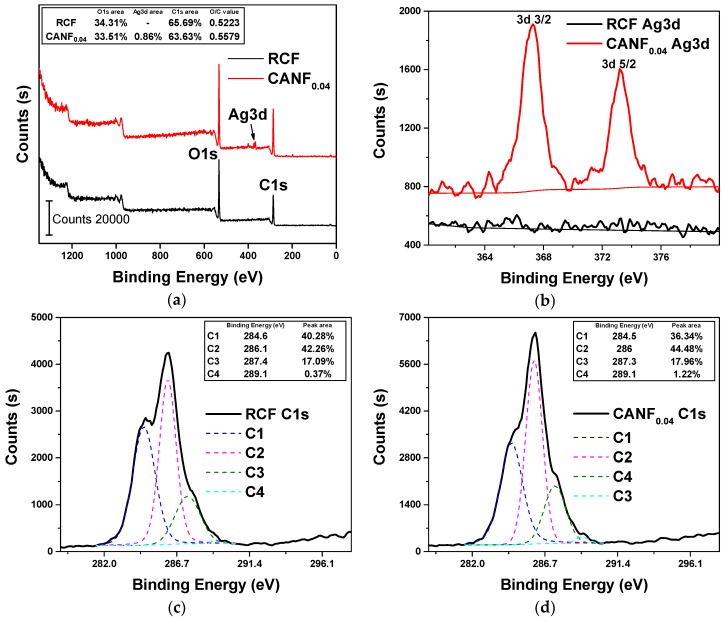
Surface element composition of RCF and CANF_0.04_: (**a**) Full scan spectrum of two films; (**b**) Scan spectrum of Ag3d peaks in two films; (**c**) Scan spectrum of C1s peaks in RCF; (**d**) Scan spectrum of C1s peaks in CANF_0.04_.

The full XPS scan results of RCF and CANF_0.04_ were shown in Figure 6a. The C1s and O1s peaks were obvious in the figure. The weak peak appeared CANF_0.04_ in the binding energy range of 380–360 was the Ag3d peak. Note that there were no obvious S and Si peaks in the spectrum indicated the less MPTS content on the film surface. This occurred because only a small amount (1% typical of filler mass) of MPTS could completely coat the nanoparticles, and most of them may be removed during the immersion process. The oxygen and carbon atomic ratios (O/C) of RCF and CANF_0.04_ surface were 0.522 and 0.558, respectively, which were close to the O/C values of common wood fibers [39]. The O/C value of RCF was slightly lower than CANF_0.04_, which indicated that the addition of Ag-NPs may introduce some oxygen-rich components on the film surface. The C1 (C–C, C–H), C2 (C–O, C=N), and C3 (N–C–O, C=O) content obtained from C1s spectrum of RCF were close to the CANF_0.04_ (Figure 6c,d), showing that the addition of Ag-NPs has little effect on the C-containing components of the film surface. C4 (–O–C=O) content of CANF_0.04_ was higher may attribute to a carboxylic acid structure produced during the reduction of Ag^+^ with DMAc similar to Ag^+^ with N,N-dimethylformamide [34,36]. The Ag content on the surface of CANF_0.04_ calculated from the Ag3d peaks was 0.86% (Figure 6b), whereas no obvious Ag3d peaks appeared on the RCF surface, suggesting that the Ag-NPs were successfully loaded on the cellulose film surface. 

**Figure A1:**
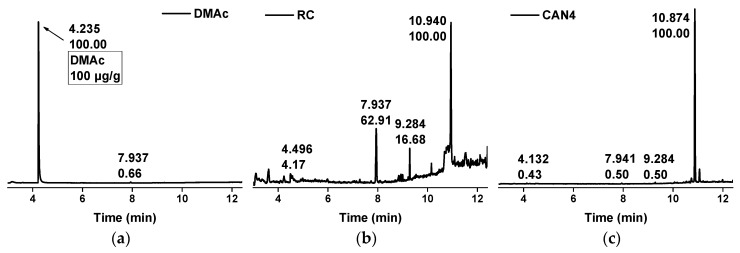
GC curves of DMAc, RCF, and CANF_0.04_: (**a**) GC curve of DMAc; (**b**) GC curve of RCF; (**c**) GC curve of CANF_0.04_.

The lithium element content of RCF and CANF_0.04_ summarized by ICP test were 0.252 mg/g and 0.241 mg/g, respectively, whereas the lithium content of 1 g film was close to that of 1 L of soft water, revealing that the low LiCl content in CANF may not harmful. The GC-MS test results (Figure A1) showed the DMAc residue in RCF and CANF_0.04_ was much lower than 100 μg/g, which limited the OEKO-TEX Standard 100. This can be attributed to the easier removal of the residual solvent in the relatively thin film (the average thickness of the films was approximately 20–40 mm). Therefore, the immersing process could effectively remove the most residual Li/DMAc system of the film, reducing potential harm to organic beings. 

**Figure 7:**
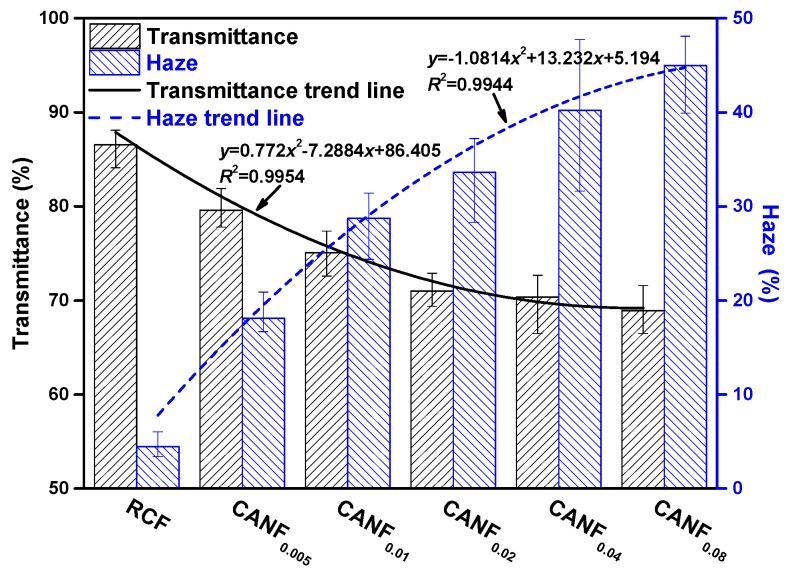
Transmittance and haze histogram of composite films.

The surface and cross section of RCF and CANF_0.04_ were dense (Figure 4c–f), and the transmittance of composite films exceeded 70% (Figure 7), corresponding to the reported high transparency cellulose-based composite films [40], indicating that the Ag-NPs-MPTS had prominent affinity with cellulose. As the Ag-NPs content increased, the more opaque Ag-NPs were dispersed uniformly in the film, resulting in a decline in film transparency. The decrease in cellulose crystallinity was also a cause of light transmittance decline. In addition, the large number of Ag-NPs promotes the increase of surface roughness of CANF, which lead to the light scattering ability enhanced, thereby the film haze was increased. The slowing trend of light transmittance and haze changed with the increase of Ag-NPs suggested the addition of too much Ag-NPs may cause a weaker effect on changed the film properties.

**Figure 8:**
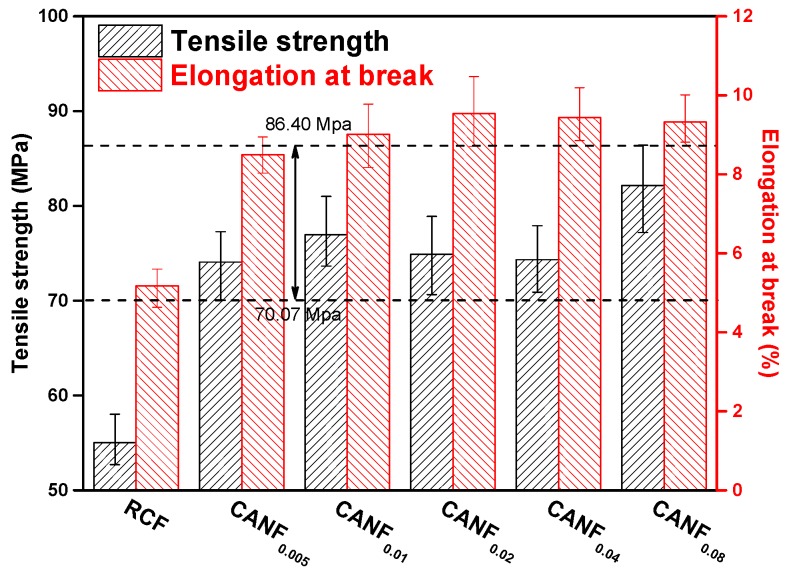
Tensile properties histogram of composite films.

Due to the good affinity between the coupled Ag-NPs and cellulose, Ag-NPs acted as an enhancer, making the tensile strength of CANF superior to RCF. The tensile strength of films from 55 MPa increased to exceed 70 MPa (reach up to 86.4 MPa), and the elongation at break increased more than 50% (Figure 8). The values were better than the silver-loaded cellulose derivative films published previously [41]. However, the tensile properties showed the complex changes with the increase in content of Ag-NPs. It may attribute to the decline of cellulose crystallinity, which reduced the order degree of cellulose chains and made it more difficult to form hydrogen bonds. Thus, the CANF were easier to stretch, and tensile properties could not be continuously increased.

**Figure 9:**
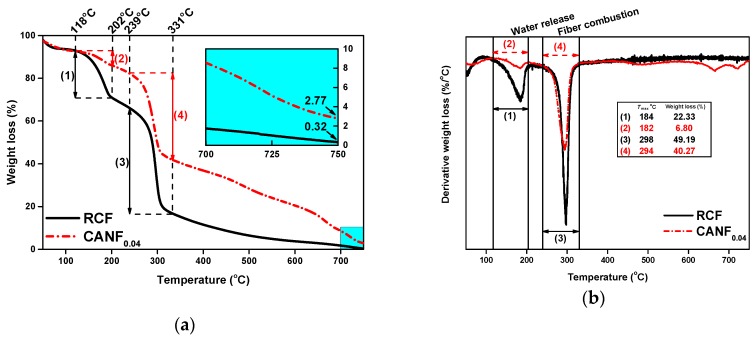
Thermal stability of composite films: (**a**) TG curves of RCF and CANF_0.04_ and (**b**) DTG curves of RCF and CANF_0.04_.

The TG and DTG curves of RCF and CANF_0.04_ showed two rapidly weight loss stages (Figure 9a,b). At 118 °C–202 °C, the weight loss of RCF and CANF_0.04_ was probably due to the water release of cellulose films. RCF and CANF_0.04_ were in hydrogel form, more bound water remained in the structure, and a large amount of bound water evaporated in this stage which may cause a rapid weight loss. Differences in weight loss for RC and CANF_0.04_ in this range may due to the swelling ability which could decrease because of Ag-NPs [42]. The weight loss in between 239 and 331 °C was mainly caused by fully combusting and pyrolyzing of cellulose. The mass loss rate difference between two films was slight, but the DTG peak of CANF_0.04_ was smaller than RCF, which reflected the coupling Ag-NPs could improve the thermal stability of cellulose film. When the temperature reached to 750 °C, the residual weight of RCF was 0.32%, and the CANF_0.04_ was 2.77%. It can be determined that the main component of the extra residue was silver, which was stable at high temperature [43]. Based on calculated by the weight of the residual silver in CANF_0.04_ and the weight of silver added in CANF_0.04_, the weight ratio of the Ag-NPs successfully loaded in CANF_0.04_ was ~61%, indicating that most of the silver was utilized, which was in line with the “sustainable” concept advocated by current research.

### 3.3. Analysis of Ag-NPs and Films’ Antibacterial Activity

The Ag-NPs solution showed strong antibacterial properties. Based on the difference in OD_600_ values, the MIC values against *E. coli* and *S. aureus* were 32 μg/mL and 64 μg/mL, respectively, revealing that the solution has been able to inhibit bacterial growth and reproduction at lower concentrations. Studies have shown that the bactericidal mechanism of Ag-NPs is mainly caused by silver clustering and anchoring to the negatively charged sites of bacterial cell wall, and then destroying the cell wall to kill bacteria [44]. However, the resistance of Gram-positive *S. aureus* with thicker cell wall to antibacterial agents is higher than that of Gram-negative *E. coli* with thinner cell wall [45]. Therefore, the antibacterial ability of Ag-NPs against *E. coli* was slightly stronger than *S. aureus*, and the MIC values of *S. aureus* were higher than *E. coli*. Based on the MBC test result, the bactericidal ratio of *E. coli* was more than 95% when the Ag-NPs concentration increased to 64 μg/mL, indicating that 64 μg/mL was the MBC value of Ag-NPs against *E. coli*, and the MBC value of Ag-NPs against *S. aureus* was 128 μg/mL can be determined as well. Lower MIC and MBC values suggest that a small amount of silver Ag-NPs could make the film antibacterial.

**Figure 10:**
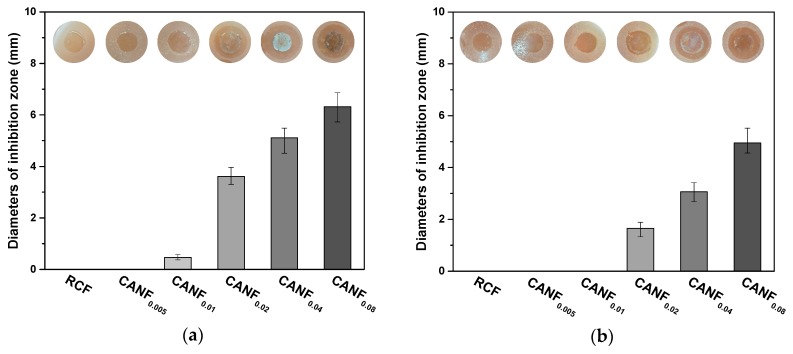
Inhibitory zone of composite films: (**a**) against *E. coli* and (**b**) against *S. aureus*.

The results of the inhibition zone test (Figure 10) showed that only CANF_0.02_, CANF_0.04_, and CANF_0.08_ have obvious circles against both bacteria. The increase of Ag-NPs concentration enlarged the Inhibition Zone of films, suggesting that the antibacterial activity of films is determined by the concentration of Ag-NPs. The inhibitory zone diameter of CANF_0.04_ and CANF_0.08_ against *E. coli* was greater than 5 mm, and the diameter against *S. aureus* was greater than 3 mm. This indicated that the Ag-NPs in composite films owned antibacterial diffusivity when the concentration reached to a certain degree. Studies have shown that Ag-NPs has cytotoxicity in vitro experiments. When the concentration was exceeded 1 mg/kg, experimental mice exhibited liver toxicity via taken orally [46]. The ICP results showed the residual silver of CANF_0.04_ diffused in NA plate was 35.3 μg, which was lower than the dose that produced cytotoxicity. The results suggest that the diffusivity of Ag-NPs may be less harmful to the organism, and cytotoxicity need to be further characterized. Thus, the composite films may have the potential to be applied in some biological fields.

Two kinds of bacteria incubated on the blank NA plate and RCF NA plate grew well (Table 1), whereas the bacteria incubated on the surface of the CANF_0.04_ did not multiply. It can be seen that the contact sterilization effect of CANF_0.04_ against both bacteria exceed 99.9%. The remarkable bactericidal effect can be attributed to the large number of Ag-NPs uniformly distributed on the surface of the CANF_0.04_ (Figure 5b), which makes it easier for the Ag-NPs attached to the bacterial cell wall when the film was in contact with bacteria. 

## 4. Conclusions

In summary, this study investigated the preparation of Ag-NPs solution using in situ reduction and one-pot synthesis of cellulose-Ag-NPs composite films. Due to the aggregation effect of Ag-NPs and the coupling effect of MPTS, the process of Ag-NPs growth and cross-linking with cellulose during the one-pot synthesis were conducted simultaneously. A uniform Cellulose-Ag-NPs composite film was successfully prepared. The CANF have better tensile properties, thermal stability, and antibacterial ability because of Ag-NPs. The large number of Ag-NPs uniformly distributed on the surface of the CANF_0.04_ makes it easier for the Ag-NPs attached to the bacterial cell wall and kill the bacteria that gives the film good antibacterial properties. In addition, the amount of residual LiCl/DMAc system in the film was low, and the amount of silver diffusion was lower than the dose that produces cytotoxicity. Therefore, the silver-loaded cellulose film obtained in the experiment may have potential for applications in food packaging and bacterial barrier.

## Figures and Tables

**Table 1:** Test results of films’ contact sterilization ability.

Sample	*E. coli*		*S. aureus*	
	Bacterial Growth ^1^	Sterilization Rate (%)	Bacterial Growth ^1^	Sterilization Rate (%)
Blank control	+	0	+	0
RCF	+	0	+	0
CANF_0.04_	-	100	-	99.9

^1^ ”+” meant growth and ”-” meant not growth.

## Appendix A

The GC curves of DMAc standard solution (100 μg/g), RCF and CANF_0.04_ obtained by GC-MS scanning as shown in the Figure A1.
